# Abnormal mechanical stress due to excessive femoral torsion is associated with dysplasia of the distal femoral epiphyseal plate and trochlea

**DOI:** 10.1302/2046-3758.1412.BJR-2025-0146.R1

**Published:** 2025-12-04

**Authors:** Lingce Kong, Chongyi Fan, Ming Li, Fei Wang, Huijun Kang

**Affiliations:** 1 Department of Orthopaedic Surgery, Third Hospital of Hebei Medical University, Shijiazhuang, China; 2 Department of Orthopaedic Surgery, Aerospace Center Hospital, Beijing, China

**Keywords:** Patellar instability, Trochlear dysplasia, Distal femoral torsion, Developmental patellofemoral dysplasia, epiphyseal plates, Distal femoral, trochlea, mechanical stress, Dysplasia, knees, epiphysis, rat model, growth plates

## Abstract

**Aims:**

The distal femoral epiphysis and epiphyseal plate are essential for skeletal morphogenesis during development. However, it is unclear how these growth mechanisms are affected by distal femoral torsion (DFT) and patellar instability. This study aimed to investigate how DFT development affects epiphyseal plate growth mechanisms.

**Methods:**

This study evaluated CT-based 3D reconstructed images of the distal femoral epiphyseal plates in 98 knees exhibiting trochlear dysplasia (50 patients). Morphological parameters including femoral anteversion, DFT, and the anatomical epicondylar axis-posterior condylar line (AEA-PCL) angle were measured to determine their relationship with epiphyseal plate development. Finite element modelling was then performed to evaluate how patellar displacement and distal femoral rotation influence epiphyseal stress in juvenile knees. A rat model that had undergone femoral rotational osteotomy was established (n = 12), and trochlear morphology (groove angle and depth) and trabecular microarchitecture (bone volume fraction, thickness, number, and separation) were compared with control specimens by micro-CT analysis at skeletal maturity.

**Results:**

Underdeveloped medial femoral epiphyseal plates were associated with excessive DFT and a large AEA-PCL angle. The medial-to-lateral epiphyseal plate ratio was inversely correlated with DFT and the AEA-PCL angle, suggesting mechanical influences on growth plate morphology. Finite element analysis revealed that medial patellar displacement and femoral external rotation decreased overall epiphyseal stress and shifted its distribution medially. Compared with control specimens, the experimental rats had significantly increased trochlear angles accompanied by reduced trochlear depth and subchondral bone loss in the medial femoral condyles and anterior medial epiphyses.

**Conclusion:**

DFT alters stress distribution across the epiphysis and epiphyseal plate, which modifies the trabecular microarchitecture in both medial femoral condyles and anterior medial epiphyses, and results in different medial-to-lateral ratio of the distal femoral epiphyseal plate which indicates the severity of trochlear dysplasia, although genetic investigations are needed to establish its causality.

Cite this article: *Bone Joint Res* 2025;14(12):1109–1122.

## Article focus

- This study focuses on the correlation between the development of the medial and lateral epiphyseal plates of the distal femur and anatomical parameters in paediatric patients with trochlear dysplasia.
- It also investigates whether changes in stress distribution across the epiphysis and epiphyseal plate, following distal femoral torsion (DFT), affect the trabecular microarchitecture in the trochlear subchondral bone and the epiphyseal region.

## Key messages

- Paediatric patients with excessive DFT have an imbalance in the development of the medial and lateral epiphyseal plates of the distal femur, as evidenced by the smaller percentage of the medial epiphyseal plate.
- Bony deformities such as patellar dislocation and DFT will cause a medial to lateral shift of stress on the distal femoral epiphysis and epiphyseal plate, and result in under-stressing of the anterior medial condyle and over-stressing of the anterior lateral condyle.
- In a rat animal model of increased femoral torsion angle, trochlear dysplasia and bone loss were present in the subchondral and epiphyseal portions of the medial femoral condyle.

## Strengths and limitations

- Using a combination of imaging measurement, finite element analysis, and animal model, this study provides clear evidence that abnormal mechanical stress due to excessive femoral torsion results in dysplasia of the distal femoral epiphyseal plate and trochlea.
- The limitations of the study include a small number of participants in the imaging measurements, a lack of generalizability across populations in the finite element analysis due to the models being constructed using data from only two independent patients, and unquantified torsion angle in the establishment of rat animal model.

## Introduction

Patients with patellar dislocation likely have morphological abnormalities of the distal femur that underlie the compromised anatomical alignment of the patellofemoral joint. Among these abnormalities, trochlear dysplasia is a predominant contributing factor.^1-3^ Previous studies have demonstrated a substantial association between distal femoral torsion (DFT) and trochlear dysplasia.^4-6^ Furthermore, biomechanical analyses indicate that internal rotation of the distal femur generates increased stress on the lateral trochlear articular surface relative to the central and medial trochlea. These altered load distributions induce mechanical adaptations in both cartilage and subchondral bone.^7-12^

The femoral epiphyseal plate is essential for longitudinal bone growth,^13^ and its distal morphology arises from regulated ossification within the secondary ossification centre. Furthermore, locomotor biomechanics profoundly influence bone development, particularly during growth phases,^14^ and the juvenile epiphysis and growth plate demonstrate mechanical sensitivity due to the pliability of developing bone tissue, including the secondary ossification centre.^15^ Clinical observations reveal that trochlear dysplasia frequently coincides with medial growth plate abnormalities in the distal femur, and has dimensional correlations to femoral trochlear morphology.^16^ Femoral anteversion may alter patellofemoral stress distribution, potentially disrupting epiphyseal ossification and subsequently modifying trochlear and condylar morphology.^17-19^ However, the relationship between growth plate dynamics and associated skeletal abnormalities, particularly DFT, remains unexplored. DFT may share developmental origins with growth plate irregularities. Furthermore, FA, patella alta, and tibial tuberosity lateralization frequently coexist with trochlear dysplasia, collectively compromising patellofemoral stability.^7,20,21^ These developmentally acquired abnormalities, termed ‘developmental patellofemoral dysplasia’, may reflect mediolateral growth plate imbalance during skeletal maturation.

Animal models of patellar dislocation have demonstrated subchondral bone changes wherein trabecular bone alterations modify femoral trochlear morphology.^22-24^ However, the relationship between trabecular bone changes in the femoral epiphysis and morphological alterations in both the femoral trochlea and its underlying subchondral bone remains unknown. Clarifying this relationship could provide critical insights into trochlear dysplasia pathogenesis.

In this study, CT imaging measurements were performed to examine the relationship between the development of medial and lateral growth plates in the distal femur and DFT, and other parameters such as femoral anteversion and the anatomical epicondylar axis-posterior condylar line (AEA-PCL) angle. Finite element models of paediatric knees with patellar displacement and DFT were constructed to analyze the shift of stress distribution across the epiphyseal plate and epiphysis. Increased femoral torsion was induced in developing Sprague-Dawley rats by femoral rotational osteotomy, and subsequent morphological changes in the femoral trochlea and epiphyseal bone mass were observed. Our hypothesis was that DFT is associated with the development of medial and lateral epiphyseal plates of distal femur, alters the stress distribution across the epiphysis and epiphyseal plate, and results in corresponding modification in the subchondral bone of the trochlea and the epiphyseal region.

## Methods

### Patient imaging measurements

**Fig. 1:**
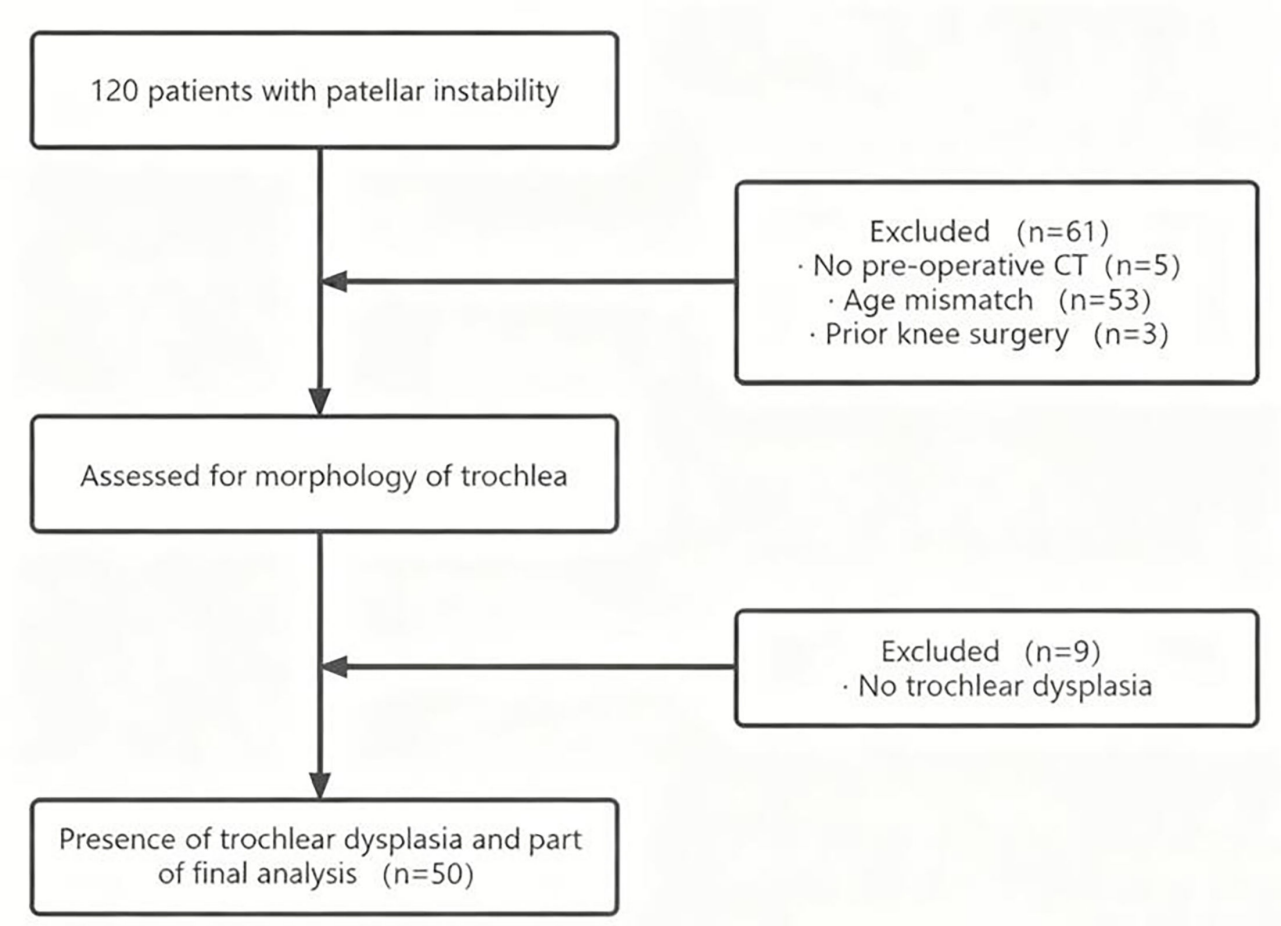
Flowchart of patient inclusion.

The ethics committee of Third Hospital of Hebei Medical University approved this study prior to its commencement, and all participants provided written informed consent. We followed the ARRIVE guidelines and completed the accompanying checklist, which has been included as Supplementary Material. From our health centre database, we retrospectively analyzed the medical records of 120 paediatric patients who underwent surgical treatment for patellar dislocation with trochlear dysplasia between January 2021 and January 2024. The diagnosis of trochlear dysplasia was confirmed through assessments of medical history, physical examination, and radiological and CT imaging. The inclusion criteria were as follows: 1) a diagnosis of trochlear dysplasia; 2) available CT imaging data and recognizable epiphyseal plate contours; 3) no history of trauma or surgery in the affected knee; and 4) patient aged 12 to 16 years. Patients who met the following criteria were excluded: 1) no preoperative CT or incomplete CT imaging of the proximal or distal trochlea; 2) age > 16 years or a closed growth plate; 3) no diagnosis of trochlear dysplasia; 4) history of trauma or surgery in the affected knee; and 5) diseases such as systemic joint laxity, rheumatoid arthritis, severe neuromuscular disease, congenital disease, connective tissue or blood disease, osteomyelitis, and bone tumour. Figure 1 shows the flowchart of patient selection. A total of 98 knees in 50 patients were included in the final cohort. The demographic data such as age, sex, affected side, and BMI were extracted directly from the medical records.

**Fig. 2:**
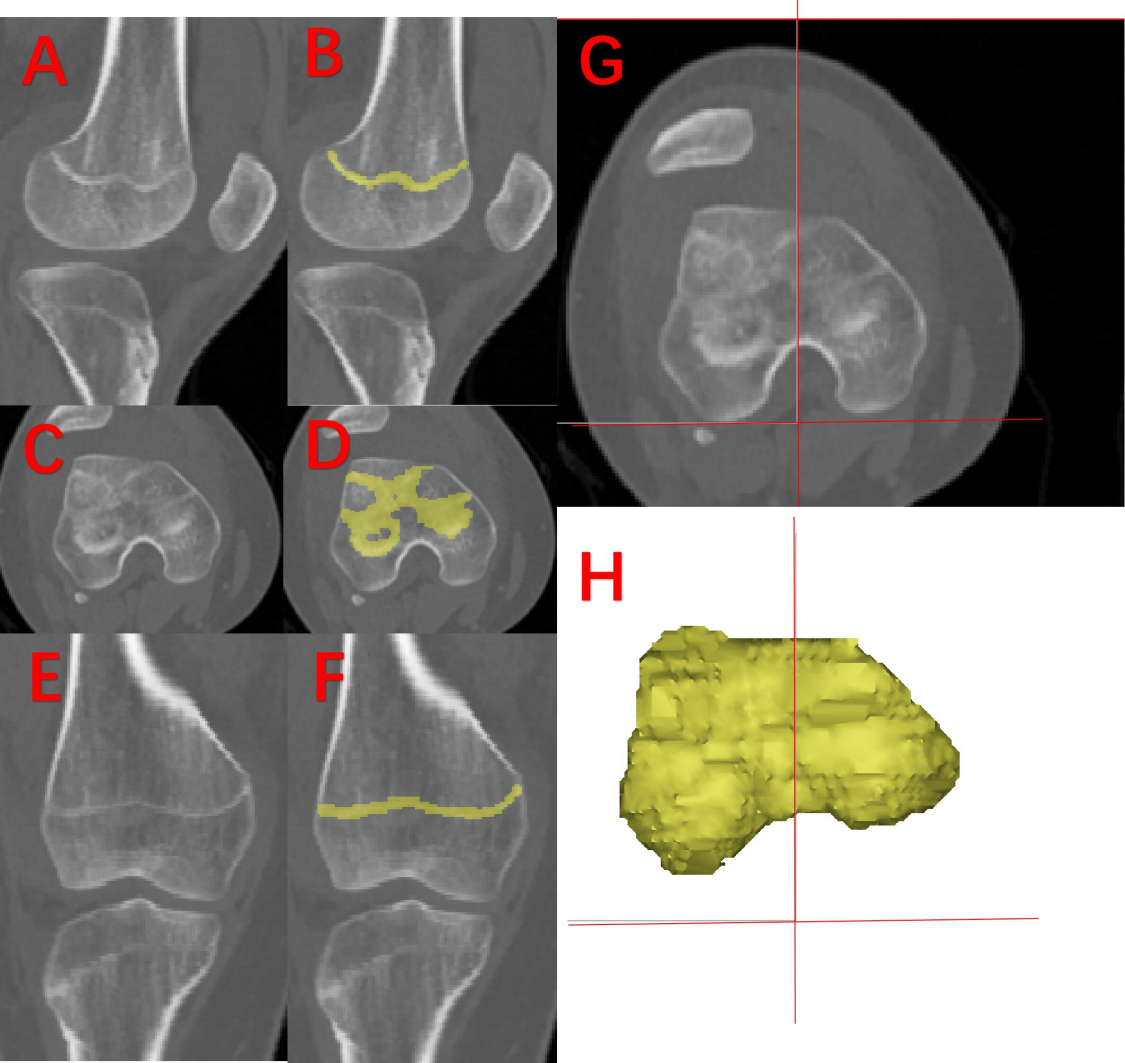
3D reconstruction of the epiphyseal plate and the division of the medial and lateral sides. a), c), and e) CT images of sagittal, axial, and coronal views. b), d), and f) Sagittal, axial, and coronal images after CT depicting the contour of the epiphyseal plate. g) and h) Schematic diagram of the reconstruction model and division of the epiphyseal plate.

**Fig. 3:**
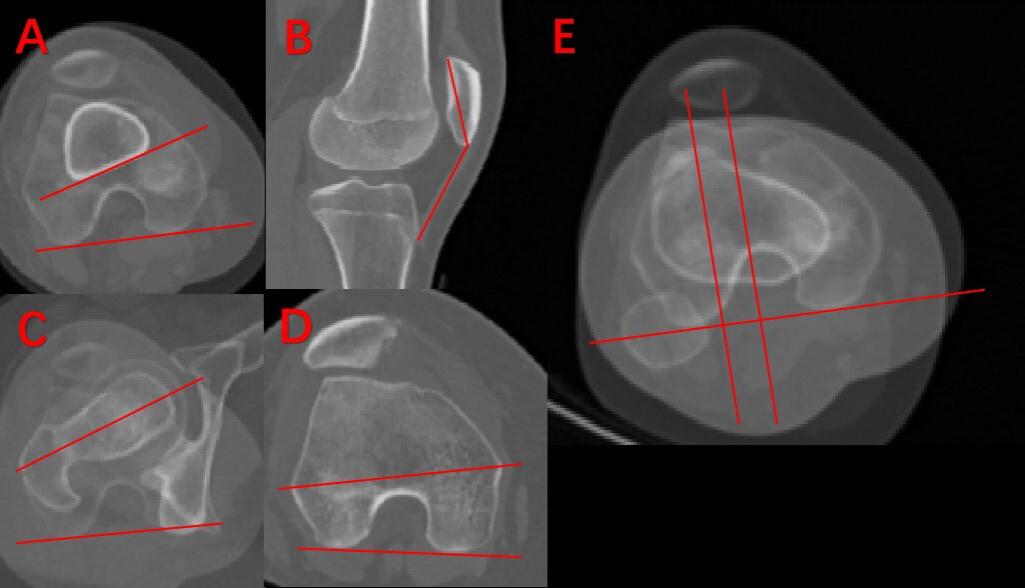
Schematic diagram of the measurement index. a) Distal femoral torsion (DFT), the angle between the posterior femoral condyle line and the posterior tangent line of the supracondylar. b) Patellar height (Insall-Salvati index), the ratio of the distance from the inferior pole of the patella to the tibial tuberosity with the longest diameter of the patella. c) Femoral anteversion. The angle between the femoral head neck line and the femoral posterior condyle line. d) Anatomical epicondylar axis-posterior condylar line (AEA-PCL). The angle between the femoral condylar axis and the posterior condyle line. e) Tibial tubercle-trochlear groove distance (TT-TG). The distance between the anterior end of the tibial tuberosity and the deepest part of the trochlear groove projected on the posterior condyle line.

3D models were reconstructed from the imaging data using Mimics 21.0 software (Materialise, Belgium), with the distal femoral epiphyseal plate visualized through the depiction method described by Kong et al.^16^ To account for variations in age and bone size, the ratio of the medial to lateral epiphyseal plate volume was calculated (Figure 2). Other assessed parameters linked to trochlear dysplasia were: FA, DFT, tibial tubercle-trochlear groove distance (TT-TG), patellar height, and AEA-PCL angle. Figure 3 illustrates the definitions and measurements of these parameters.

Patients were categorized based on CT measurements of specific parameters. For patellar height, an Insall-Salvati index between 0.8 and 1.2 was considered the normal group, between 1.2 and 1.5 was considered the high group, and > 1.5 was considered the super-high group. For TT-TG, < 20 mm was considered the normal group and > 20 mm was considered the high-value group. For FA, < 25° was considered the normal group and > 25° was considered the high-value group. For DFT, < 15° was considered the normal group and > 15° was considered the high-value group. For the AEA-PCL angle, < 6° was considered the normal group and > 6° was considered the high-value group. The thresholds for the division of group were obtained from previous studies.^8,25,26^

### Finite element analysis

The knee models were created based on the knee of a girl with patellar dislocation, excessive DFT, and trochlear dysplasia, and the knee of a control subject matched for sex, age, and BMI. Knee CT scans were reconstructed with Mimics 21.0 using threshold segmentation and 3D modelling algorithms to generate anatomical structures including the femur, tibia, patella, fibula, epiphysis, and growth plate, with subsequent export as STL files. These files were processed in Geomagic 2021 (3D Systems, USA) for mesh optimization through uniform refinement, surface smoothing, and curvature adjustment. Ligament, meniscus, and cartilage models derived from CT and MRI underwent identical geometrical processing. The epiphysis and femur were segmented, realigned, and contoured to delineate the growth plate before final assembly in SolidWorks 2021 (Dassault Systèmes, USA). Two distinct 3D anatomical models were created, with subsequent modifications to simulate patellar displacement and femoral rotational deformities.

**Fig. 4:**
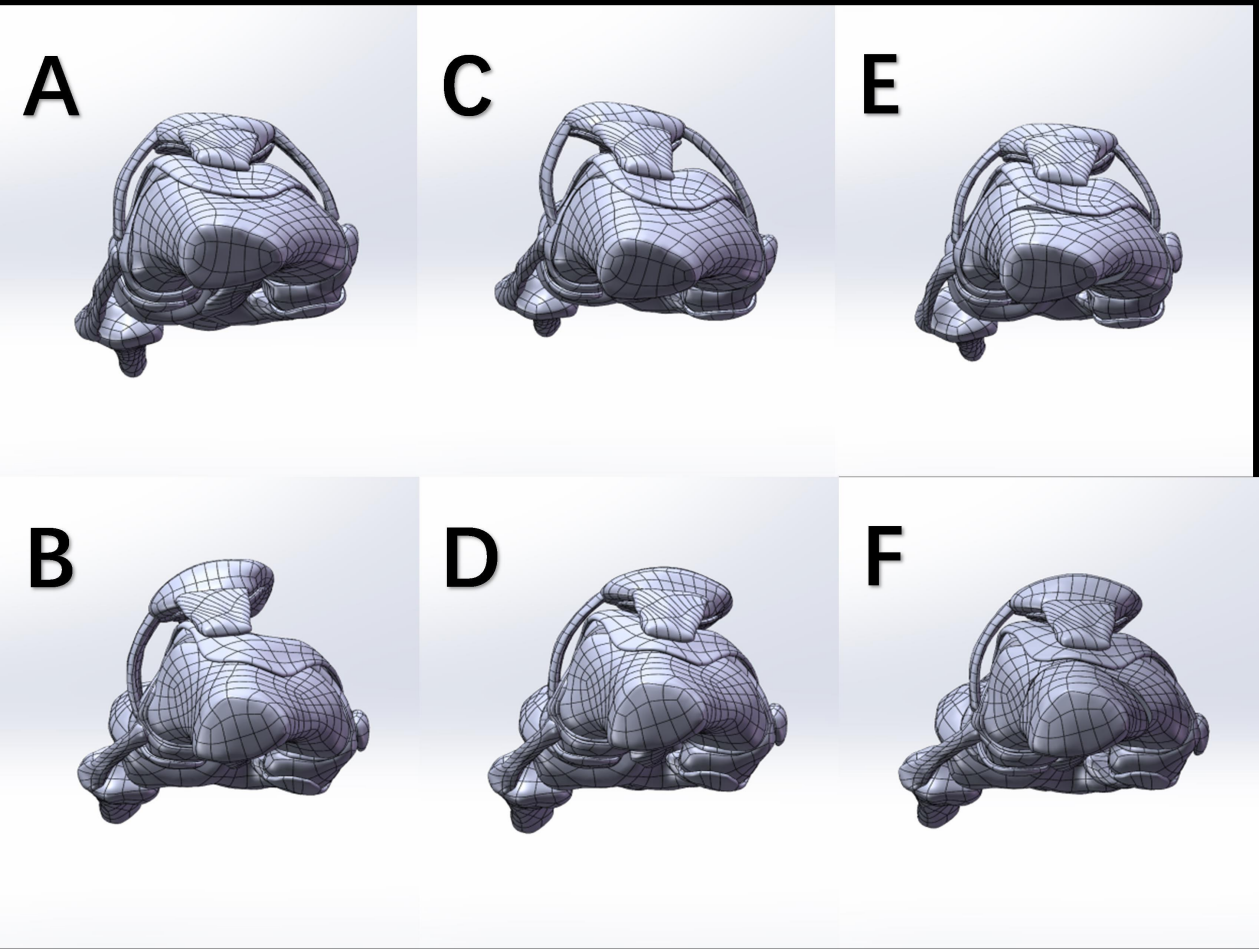
Schematic diagram of the top view of each finite element model. a) Normal development of knee joint. b) Trochlear dysplasia with patellar dislocation. c) The patella of normal developing knee joint moves laterally by 10 mm. d) Trochlear dysplasia knee patella moved medially 10 mm. e) The distal femur of the normal development knee was rotated internally by 10°. f) The distal femur of the trochlear dysplasia knee was rotated externally by 10°.

Finite element mesh models of the femur, tibia, articular cartilage, and ligaments were constructed using ANSA (BETA CAE Systems, Greece), with material properties for each component detailed in Table I. Two baseline knee models were modified through medial/lateral patellar displacement (10 mm) and distal femoral rotational osteotomy (10°) to alter anatomical parameters. After these adjustments, stress distribution changes in the epiphysis were analyzed at 30° flexion. A total of six models were established to represent: 1) normal knee; 2) trochlear dysplasia with patellar dislocation; 3) 10 mm lateral patellar movement in the normal knee; 4) 10 mm medial patellar movement in knee with trochlear dysplasia; 5) 10° internal rotation of the distal femur in the normal knee; and 6) 10° external rotation of the distal femur in the knee with trochlear dysplasia (Figure 4). A 3D four-noded tetrahedral element was used for modelling. To ensure the convergence of displacement and stress, the mesh sizes of the stress region and contact region in the model were refined to a minimum of 1 mm, and the mesh independency was checked. The convergence tolerance of the contact analysis was set to 1%. The number of mesh nodes was 2,019,438 to 2,229,584, while the number of elements was 1,384,831 to 1,536,022.

**Table I.** Material parameters.

Material	Elastic modulus (Mpa)	Poisson’s ratio
Epiphysis	17,000	0.3
Cancellous bone	900	0.3
Cortical bone	17,000	0.3
Epiphyseal plate	35	0.3
Anterior cruciate ligament	10.14	0.3
Posterior cruciate ligament	16.9	0.3
Lateral collateral ligament	7.488	0.3
Medial collateral ligament	7.488	0.3
Patellar ligament	225	0.3
Patellofemoral ligament	260	0.3
Meniscus	27.5	0.33
Articular cartilage	15	0.46
Quadriceps	304	0.46

The ends of the distal tibia and fibula were restrained. A 400 N tensile force was applied at the upper edge of the quadriceps to simulate muscle lifting, and a 300 N vertical load was added at the upper end of the proximal femur to simulate half the body weight of a normal human.

The von Mises stress distribution in the epiphysis appeared in both the frontal and axial views. The axial projection specifically illustrated stress patterns across the growth plate. Colour gradients in the nephograms quantified stress magnitudes within each finite element model, providing immediate visualization of localized stress concentrations.

### Animal experiments

All animal procedures followed the National Research Council’s Guide for the Care and Use of Laboratory Animals.^27^ A total of 15 three-week-old female Sprague-Dawley rats were housed in five groups, provided with ad libitum access to water and standard chow, and kept at a room temperature of 18°C to 25°C. After one week of acclimatization, we performed surgical interventions. Preoperative assessments were performed to confirm normal knee mobility and gait in all animals. The left knees were designated as the experimental limbs that underwent femoral rotational osteotomy, while the right knees were left untreated as controls. Rats achieve skeletal maturity by 12 weeks of age;^28^ therefore, femoral epiphyseal development was evaluated eight weeks postoperatively.

**Fig. 5:**
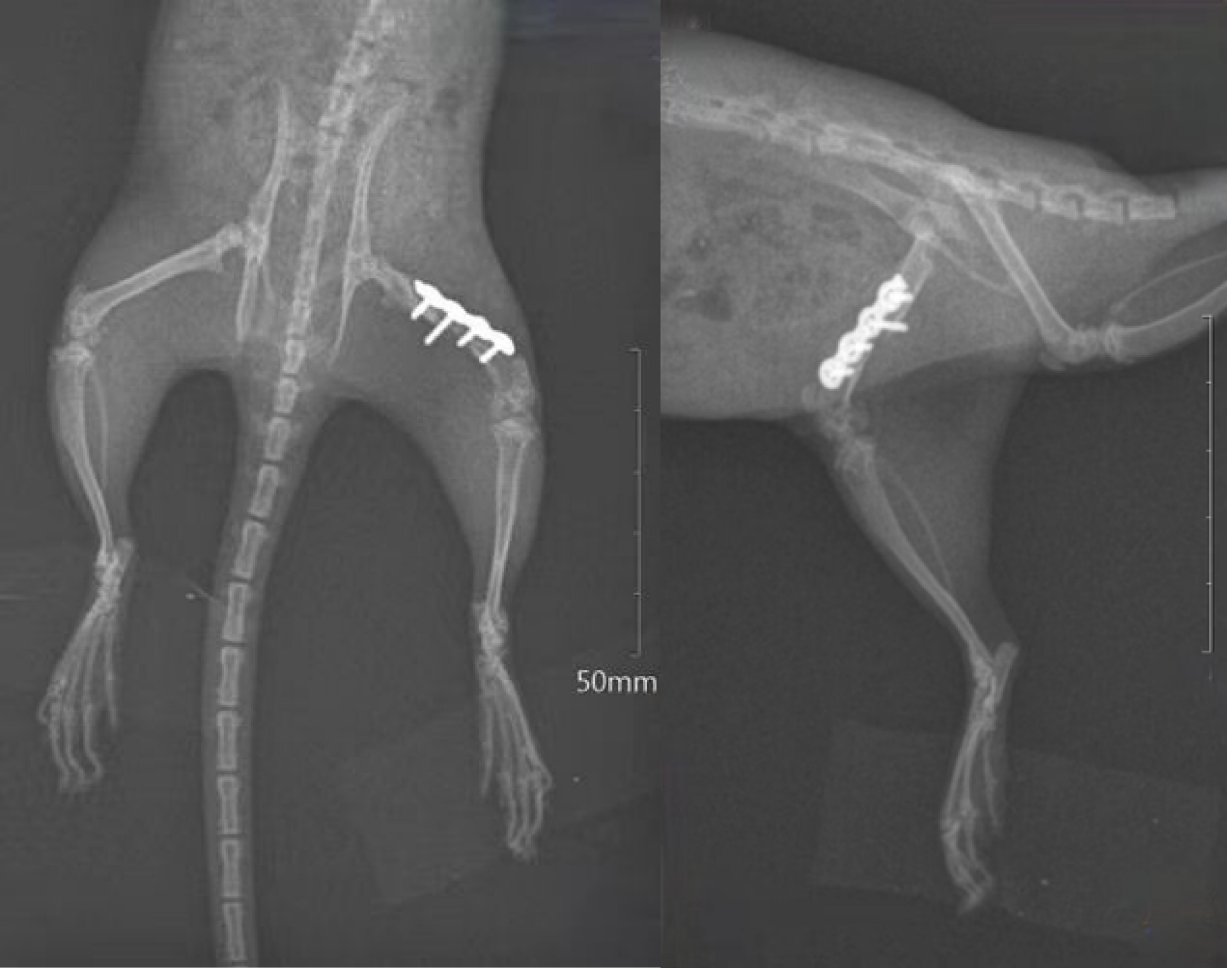
Coronal and sagittal radiographs of one rat two weeks after femoral rotational osteotomy.

The animals were fasted for 12 hours and anaesthetized with an intraperitoneal injection of 2% pentobarbital sodium (50 mg/kg). We placed the rats in a supine position and performed standard skin preparation, iodine disinfection, and draping. A 3 cm longitudinal incision was made along the lateral midline of the thigh with the left knee slightly flexed. The skin, subcutaneous tissue, and fascial layer were sequentially incised to expose the fascial gap between the vastus lateralis and biceps femoris muscles. Further dissection revealed the femoral shaft cortex. The transverse osteotomy site was selected at either the midpoint or distal portion of the femoral shaft, based on the internal fixation length. A four-hole locking plate was positioned along the posterior edge of the femoral shaft, with two locking screws inserted to secure the distal osteotomy line. Following stable fixation of the distal fragment and plate, we fixed the proximal fragment and internally rotated the distal end to maintain torsional displacement. The goal was to increase the distance between the posterior ridge of the distal and proximal femur by approximately 1 to 2 mm (about a 20° increase in torsion).^24^ Subsequently, two locking screws were inserted to secure the proximal femur to stabilize the osteotomy. The osteotomy line was maintained parallel throughout rotation. Passive knee flexion and extension were performed postoperatively to assess the patellar tracking. Patellar lateral displacement or dislocation may occur at the terminal stage of extension. After irrigation with physiological saline, the surgical site was closed in layers using standard fascial, subcutaneous, and skin sutures (Figure 5). Each animal was housed individually and received cephalosporin antibiotics and non-steroidal anti-inflammatory drugs in their feed for five days postoperatively to prevent infection and manage pain. Elizabethan collars were fitted to prevent self-inflicted trauma at the surgical site. Rats progressively resumed their normal activity as the postoperative pain subsided, with no movement restrictions imposed. After an observation period of eight weeks, the rats were euthanized via pentobarbital sodium overdose. Two rats died due to extensive surgical trauma on postoperative day 2, leaving 13 survivors. During tissue harvest at eight weeks postoperatively, one rat exhibited severe femoral infection and was excluded from the analysis. The remaining animals underwent soft-tissue dissection, with the intact femora collected for macroscopic evaluation. Specimens were fixed in 10% formalin for subsequent micro-CT scanning (n = 12).

All samples were fixed and prepared for micro-CT scanning using the following parameters: 200 μA tube current, 85 kV voltage, 10.03 μm isotropic resolution, 384 ms exposure time, and 180° rotation. A manufacturer-supplied phantom was scanned under identical conditions for calibration. The raw scan data were reconstructed into cross-sectional images. These images were then analyzed using Mimics 21.0 software for quantitative measurements.

**Fig. 6:**
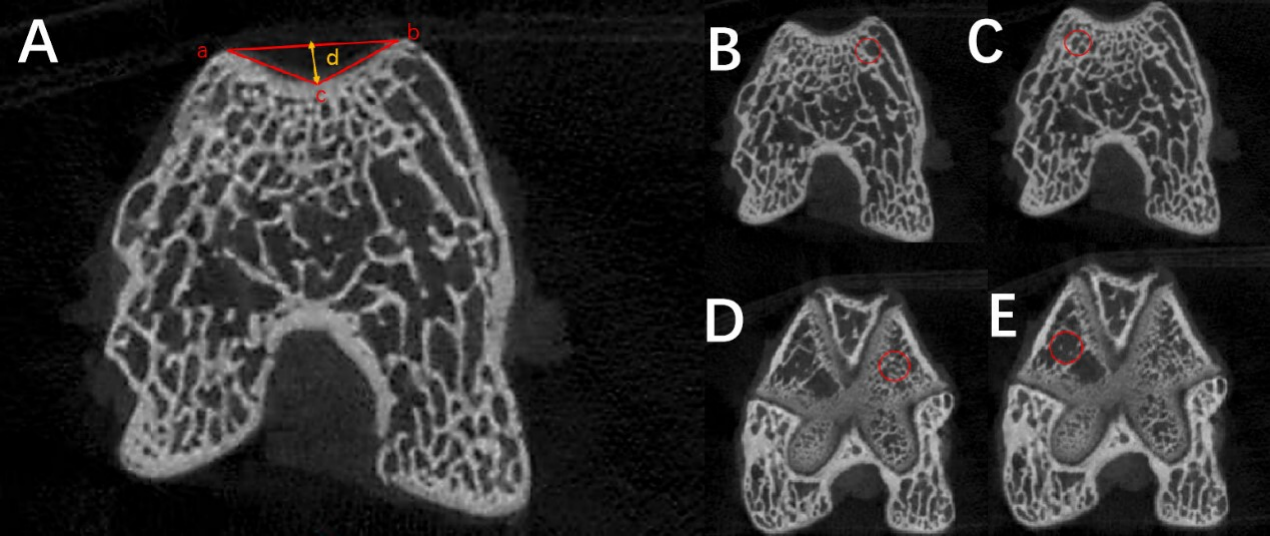
Trochlear groove depth, trochlear groove angle, and region of interest area selection diagram. a) Angle a-c-b represents the trochlear groove angle, and d represents the trochlear groove depth. b) and c) Region of interest in the medial/lateral trochlear subchondral bone. d) and e) Region of interest in the anterior lateral/medial epiphysis.

The original images were reconstructed and regions of interest (ROIs) were selected using NRecon software (version V1.7.4.2, Bruker Corporation, Germany). A preview of the reconstructed images was performed prior to final reconstruction. Image artifacts were minimized by adjusting the following reconstruction parameters: smoothing = 5, beam-hardening = 8, and ring artifacts = 25%. CT Analyser software (version 1.20.3.0; Bruker Corporation) was used for the ROI analysis. ROIs were selected from the proximal femoral trochlea, including areas adjacent to the medial and lateral condyles beneath the cartilage, where two cylindrical regions (1 mm diameter, 0.5 mm height) were sampled. Additional ROIs were taken from the ossified region near the femoral epiphyseal plate (within 1 mm), with two cylindrical samples (1 mm diameter, 0.5 mm height) collected near the anterior medial and lateral epiphyseal plate (Figure 6). Using uniform parameters, the software calculated tissue properties including bone mineral density (BMD), bone volume fraction (BV/TV), trabecular number (Tb.N), trabecular thickness (Tb.Th), and trabecular separation (Tb.Sp).

The proximal plane of the femoral trochlea was first identified, typically through the articular cartilage layer covering the trochlear surface, which served as the reference plane. A CT plane 5 mm distal to this proximal plane was then used as the designated measurement plane for assessing the trochlear groove angle. This angle was defined by two lines: one connecting the peak of the medial femoral condyle to the lowest point of the trochlear groove, and another linking the peak of the lateral condyle to the same reference point.^29^ Trochlear depth was measured as the perpendicular distance from the lowest point of the trochlear groove to the line connecting the medial and lateral femoral condyle peaks (Figure 6).

### Statistical analysis

Continuous variables are reported as mean (SD), while categorical variables are presented as frequencies and percentages. Data normality was assessed using the Kolmogorov-Smirnov test. Independent-samples *t*-tests were used to compare normally distributed variables between the study and control groups, whereas one-way analysis of variance (ANOVA) was used to analyze differences between more than two groups. Pearson correlation coefficients were used to quantify the relationships between measured parameters. Paired *t*-tests were used to evaluate within-group differences, while the Mann-Whitney U test was used to compare non-normally distributed data between groups. All analyses were conducted using SPSS 24.0 (IBM, USA), with statistical significance set at p < 0.05. Two experienced surgeons (LK, HK) used the same standard to independently measure data twice with a four-week interval. The intraclass correlation coefficients (ICCs) were calculated to determine whether the correlation was good (ICC 0.6 to 0.8) or excellent (ICC 0.8 to 1.0). Sample size estimation was conducted with G*Power 3 (Heinrich Heine University, Germany). An a priori power analysis for independent-samples *t*-tests with α < 0.05 and 80% power yielded a minimum requirement of 40 knees. Subsequent correlation analysis indicated a necessary sample of 64 knees.

## Results

### Patient imaging measurements

The study cohort included 98 knees in 50 patients with trochlear dysplasia. Table II summarizes the demographic characteristics. The ICC results were from 0.853 to 0.943 with excellent reliability. The measurements from the first observer were used for further analysis.

**Table II.** Patient characteristics.

Characteristic	Data
Mean age, yrs (SD)	14.2 (1.91)
Sex (female:male), n	40:10
Side (left:right), n	49:49
Mean BMI, kg/m^2^ (SD)	24.0 (4.2)

When patients were grouped by patellar height, TT-TG, and FA, the medial-to-lateral distal femoral epiphyseal plate ratio showed no significant differences between the high- and low-value groups. However, there was a significantly lower medial epiphyseal plate volume percentage in patients with large DFT than in those with small DFT (p = 0.012, independent-samples *t*-test). Similarly, the group with a large AEA-PCL angle exhibited reduced medial epiphyseal plate volume percentages compared with those with a small AEA-PCL angle (p < 0.001, independent-samples *t*-test) (Table III). Pearson correlation analysis identified significant associations between DFT, AEA-PCL angle, and the medial-to-lateral distal femoral epiphyseal plate ratio (p < 0.001), along with a significant correlation between DFT and FA (p < 0.05) (Table IV).

**Table III.** Ratio of medial and lateral epiphyseal plates of distal femur in different groups.

Parameter	Group	Mean epiphyseal ratio, % (SD)	p-value
Insall-Salvati index	Normal (n = 34)	84.60 (7.10)	0.138*
	High-value (n = 51)	86.03 (5.56)	
	Super high-value (n = 13)	87.73 (7.61)	
TT-TG	Normal (n = 46)	85.17 (6.11)	0.397†
	High-value (n = 52)	86.28 (6.71)	
FAA	Normal (n = 45)	86.31 (6.13)	0.440†
	High-value (n = 53)	85.30 (6.69)	
DFT	Normal (n = 36)	87.89 (5.76)	0.012†
	High-value (n = 62)	84.52 (6.51)	
AEA-PCL	Normal (n = 52)	89.36 (5.20)	< 0.001†
	High-value (n = 46)	81.70 (5.15)	

* One-way analysis of variance.

† Independent-samples *t*-test.

AEA-PCL, anatomical epicondylar axis-posterior condylar line; DFT, distal femoral torsion; FAA, femoral anteversion angle; TT-TG, tibial tubercle-trochlear groove distance.

**Table IV.** Pearson correlation analysis.

Parameter	FAA	DFT	AEA-PCL	TT-TG	Insall-Salvati index	Epiphyseal ratio
FAA	1					
DFT	0.244*	1				
AEA-PCL	0.067	0.622†	1			
TT-TG	0.053	-0.089	-0.001	1		
Insall-Salvati index	0.023	0.132	0.168	0.093	1	
Epiphyseal ratio %	-0.111	-0.503†	-0.483†	-0.02	0.073	1

All p-values were measured using Pearson correlation coefficients.

* p < 0.05.

† p < 0.01.

AEA-PCL, anatomical epicondylar axis-posterior condylar line; DFT, distal femoral torsion; FAA, femoral anteversion angle; TT-TG, tibial tubercle-trochlear groove distance.

### Von Mises stress nephogram of the epiphysis

**Fig. 7:**
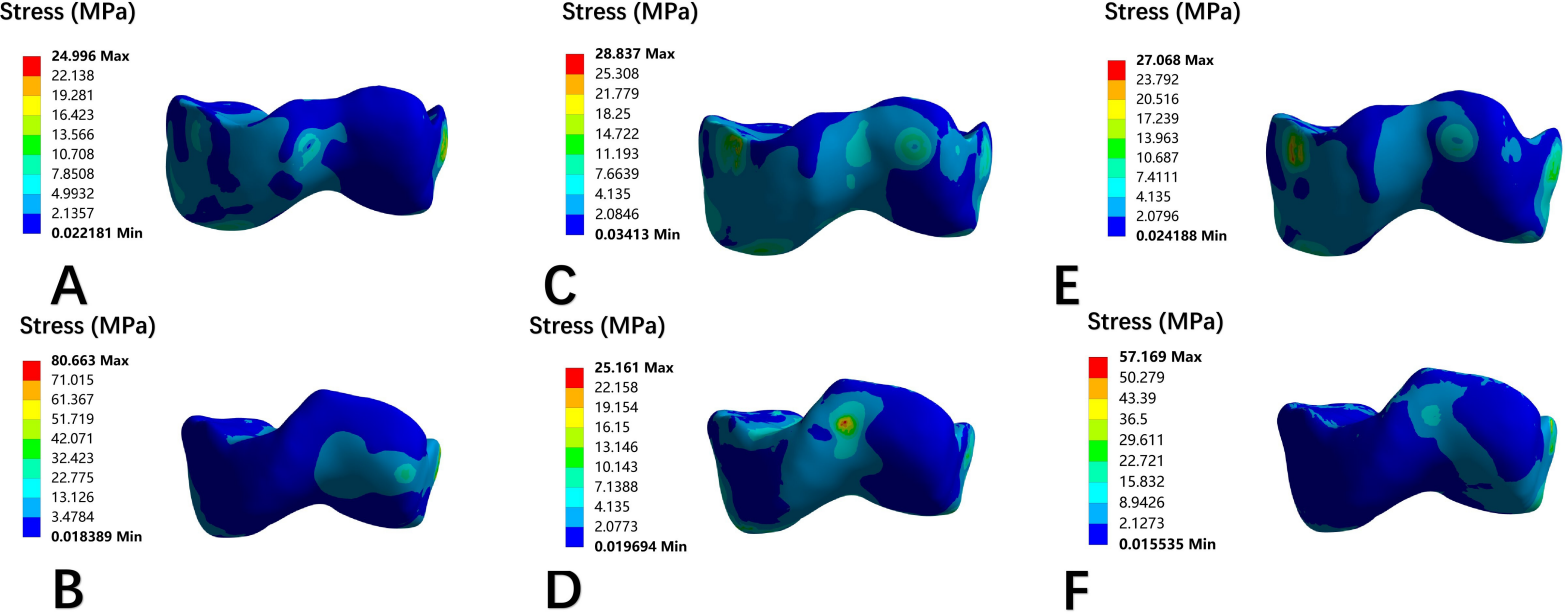
Front view nephogram of epiphyseal stress distribution of each model. a) Normal development of knee joint. b) Trochlear dysplasia with patellar dislocation. c) The patella of normal developing knee joint moves laterally by 10 mm. d) Trochlear dysplasia knee patella moved medially 10 mm. e) The distal femur of the normal development knee was rotated internally by 10°. f) The distal femur of the trochlear dysplasia knee was rotated externally by 10°.

**Fig. 8:**
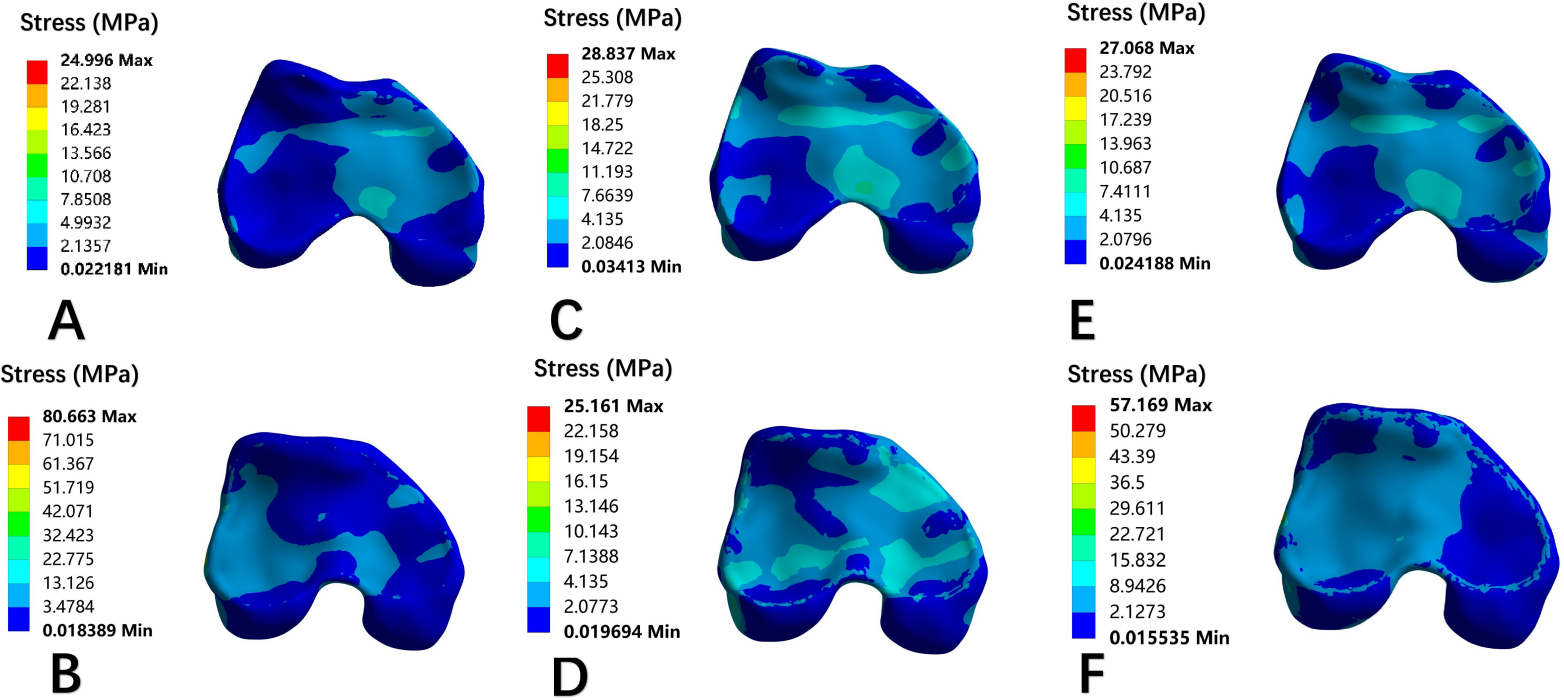
Top view nephogram of epiphyseal stress distribution of each model. a) Normal development of knee joint. b) Trochlear dysplasia with patellar dislocation. c) The patella of normal developing knee joint moves laterally by 10 mm. d) Trochlear dysplasia knee patella moved medially 10 mm. e) The distal femur of the normal development knee was rotated internally by 10°. f) The distal femur of the trochlear dysplasia knee was rotated externally by 10°.

Stress distribution on epiphyseal plate for each model was present in frontal view (Figure 7) and axial view (Figure 8). The peak stress values derived from the colour scale are shown in Table V.

**Table V.** Peak value of colour scale reading of stress nephogram of each model.

View	Model 1	Model 2	Model 3	Model 4	Model 5	Model 6
Front view of epiphysis	10.708 MPa	32.423 MPa	14.722 MPa	25.161 MPa	13.963 MPa	15.832 MPa
Top view of epiphysis	7.851 MPa	13.126 MPa	7.664 MPa	7.139 MPa	7.411 MPa	8.946 MPa

Model 1, normal knee development; Model 2, trochlear dysplasia with patellar dislocation; Model 3, 10 mm lateral patellar movement in the normal developing knee; Model 4, 10 mm medial patellar movement with trochlear dysplasia; Model 5, 10° internal rotation of the distal femur in the normal developing knee; Model 6, 10° external rotation of the distal femur in the knee with trochlear dysplasia.

After lateral patellar dislocation and internal rotation of the distal femur in the control group, the epiphyseal mechanical stress shifted from medial to lateral in the frontal plane, with peak stress increasing by 37.5% and 30.4%, respectively. The axial view demonstrated stress redistribution from the medial condyle to the anterolateral condyle, although the peak stress values did not change significantly.

In the trochlear dysplasia model with patellar dislocation, mechanical stress in the epiphysis shifted laterally to centrally in the frontal view after medial patellar displacement and external femoral rotation, with resultant decreases in peak stress by 22.4% and 51.2%, respectively. In the axial view, the stress distribution transitioned from lateral to medial, with peak stress reductions of 45.6% and 31.8%, respectively. The stress distribution of the medial patellar displacement model was better than that of the external femoral rotation model.

### Changes in the trochlea and epiphysis in rats

**Fig. 9:**
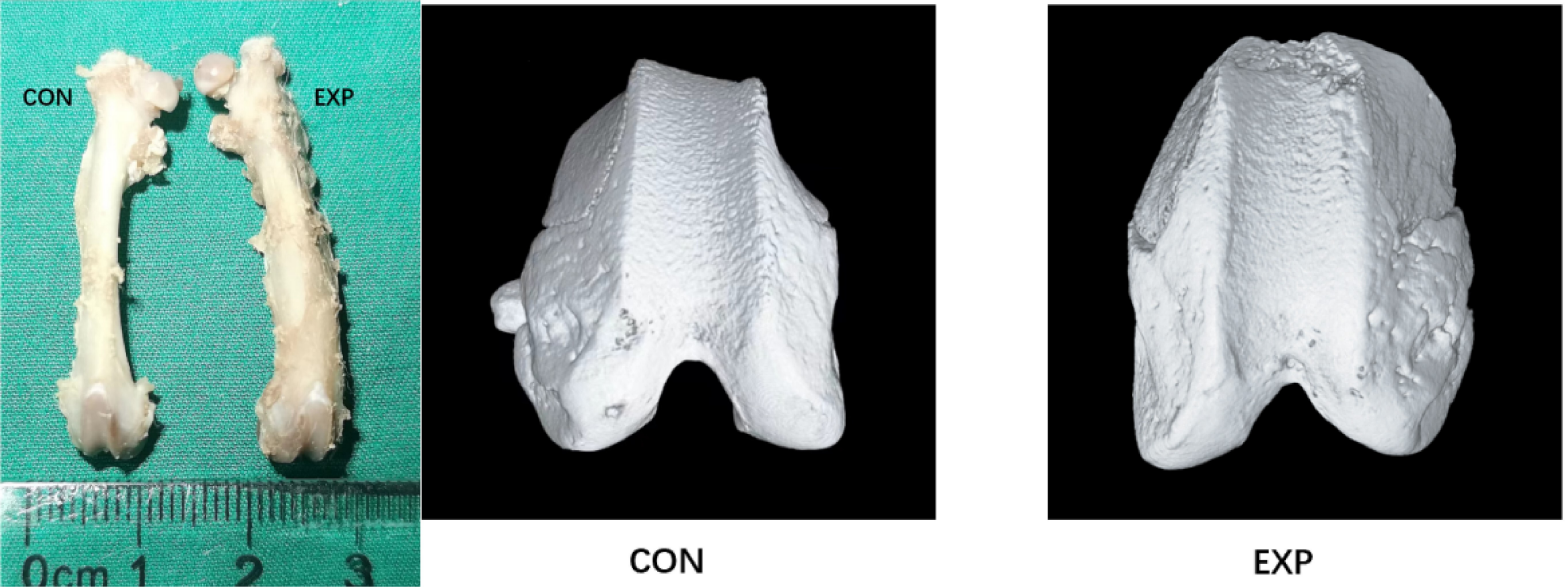
Macroscopic comparison of the whole femur between the experimental group (EXP) and the control group (CON) shows that the femoral torsion is significantly increased. The results of micro-CT found that the experimental group exhibited significantly flatter and wider trochlear groove.

A total of 12 rats completed the experimental protocol. After the eight-week observation period, two rats developed substantial dislocation during knee extension attempts, whereas the remaining ten showed no dislocation but demonstrated lateral displacement during extension. Gross observation revealed that the experimental group exhibited wider and shallower trochlear morphology than the control group, consistent with trochlear dysplasia (Figure 9). The trochlear groove angle was significantly increased and the groove depth was significantly decreased in the experimental group compared with the control group, confirming the presence of trochlear dysplasia in the experimental cohort (Table VI).

**Table VI.** Trochlear morphology between the experimental group and the control group.

Parameter	Control group	Experimental group	p-value*
Mean sulcus angle, ° (SD)	129.65 (3.37)	140.36 (3.79)	< 0.001
Mean sulcus depth, mm (SD)	0.99 (0.08)	0.71 (0.05)	0.001

* Paired *t*-test.

Micro-CT measurements revealed distinct BV/TV values between groups in the medial proximal trochlea (p = 0.003), but not in the lateral proximal trochlea (p = 0.150). The experimental group exhibited a significantly lower Tb.N than the control group in the medial proximal trochlea (p = 0.002), but not in the lateral trochlea (p = 0.167). The experimental group had a significantly greater Tb.Sp than the control group in the medial proximal trochlea (p < 0.001), but not in the lateral trochlea (p = 0.343, all paired *t*-test). The BMD and Tb.Th demonstrated only minor intergroup variations (Table VII).

**Table VII.** Micro-CT analysis results of the experimental group and the control group.

Parameter	Region	Control group	Experimental group	p-value*
BMD (g/mm^3^)	Anterior-medial epiphysis	0.532 (0.078)	0.383 (0.801)	0.003
	Medial of proximal trochlear	0.446 (0.621)	0.449 (0.264)	0.905
	Anterior-lateral epiphysis	0.531 (0.050)	0.481 (0.091)	0.100
	Lateral of proximal trochlear	0.474 (0.022)	0.429 (0.098)	0.247
BV/TV (%)	Anterior-medial epiphysis	79.042 (16.261)	45.431 (13.942)	0.003
	Medial of proximal trochlear	52.391 (4.479)	43.918 (5.491)	0.003
	Anterior-lateral epiphysis	75.039 (9.412)	59.968 (16.034)	0.032
	Lateral of proximal trochlear	55.899 (6.149)	47.650 (12.927)	0.150
Tb.Th (mm)	Anterior-medial epiphysis	0.167 (0.038)	0.134 (0.320)	0.024
	Medial of proximal trochlear	0.139 (0.067)	0.117 (0.201)	0.101
	Anterior-lateral epiphysis	0.147 (0.139)	0.144 (0.024)	0.785
	Lateral of proximal trochlear	0.141 (0.006)	0.142 (0.105)	0.866
Tb.N (1/mm)	Anterior-medial epiphysis	4.924 (0.443)	3.546 (0.867)	0.046
	Medial of proximal trochlear	3.735 (0.367)	3.059 (0.264)	0.002
	Anterior-lateral epiphysis	5.274 (0.321)	4.738 (0.496)	0.121
	Lateral of proximal trochlear	3.884 (0.347)	3.325 (0.853)	0.167
Tb.Sp (mm)	Anterior-medial epiphysis	0.081 (0.021)	0.166 (0.049)	0.011
	Medial of proximal trochlear	0.181 (0.018)	0.250 (0.239)	< 0.001
	Anterior-lateral epiphysis	0.095 (0.017)	0.118 (0.042)	0.180
	Lateral of proximal trochlear	0.179 (0.277)	0.202 (0.423)	0.343

All data shown as mean (SD).

* Paired *t*-test.

BMD, bone mineral density; BV/TV, bone volume fraction; Tb.N, trabecular number; Tb.Sp, trabecular spacing; Tb.Th, trabecular thickness.

The medial region of the distal femoral epiphysis showed lower Tb.Th (p = 0.024) and lower Tb.N (p = 0.046) in the experimental group. The Tb.Sp measurements revealed significant bone loss in the anterior medial region (p = 0.011). In contrast, the anterior lateral region exhibited comparable Tb.Th (p = 0.785) and non-significant differences in Tb.N (p = 0.121) and Tb.Sp (p = 0.180, all paired *t*-test). The results align with the trabecular bone structural pattern in the proximal medial and lateral femoral trochlea, which revealed pronounced bone loss in the medial proximal trochlear and anteromedial epiphyseal regions, but no detectable bone loss in the lateral areas (Table VII).

## Discussion

The most important finding was that patients with trochlear dysplasia with a greater DFT had a smaller medial distal femoral epiphyseal plate. Moreover, the AEA-PCL angle was correlated with asymmetric growth of the medial and lateral epiphyseal plates. Finite element modelling has shown that trochlear dysplasia, patellar dislocation, and DFT alter the stress distribution across the distal femoral epiphysis. In the rat model of increased femoral torsion induced by femoral rotation osteotomy, patellar dislocation and trochlear dysplasia were present, with a significantly greater trochlear groove angle and shallower trochlear groove depth than the normal group. Bone loss was observed simultaneously in the medial subchondral bone beneath the femoral trochlea and the medial epiphysis. Combined with imaging measurement, finite element analysis, and animal model, the present study provides clear evidence that abnormal mechanical stress due to excessive femoral torsion results in dysplasia of the distal femoral epiphyseal plate and trochlea.

The role of femoral anteversion in the recurrent patellar dislocation has been well documented.^24,30^ Furthermore, DFT is the primary cause of excessive femoral anteversion.^5,8^ In our subgroup analysis, patients with excessive DFT exhibited greater dysplasia in the medial epiphyseal plate of the distal femur. The observed morphological alterations may stem from imbalanced development in the medial and lateral epiphyseal plates, which may influence femoral torsion in reverse. As epiphyseal development determines both long bone length and skeletal shape, these findings align with a previous study that reported a link between trochlear dysplasia and medial epiphyseal plate abnormalities, which may correlate with medial femoral condyle dysplasia.^16^ Additionally, patients with patellar instability have altered lower limb length proportions, indicating that trochlear dysplasia influences epiphyseal plate development and consequently affects final limb length.^31^ Our results reveal a significant correlation between epiphyseal development and femoral torsion, offering new perspectives on the aetiology of excessive DFT. Asymmetric physeal growth corresponds with DFT, indicating that supracondylar derotational osteotomy may be the preferred surgical intervention for patellar dislocation.^4,5^

Previous studies have shown that patients with trochlear dysplasia have shorter anterior medial and posterior lateral condyles, accompanied by a larger AEA-PCL angle.^8,32,33^ In our study, the medial epiphyseal plate was more poorly developed in patients with a larger AEA-PCL angle and greater DFT. The poorly developed medial epiphyseal plate may drive condylar development posteriorly, while the more developed lateral plate appears to propel the lateral condyle forward. The adult trochlear morphology emerges during growth from the anterior portion of the distal femoral epiphyseal plate, which arises anterior to the plate and extends proximally toward the anterior cortex.^34^ The epiphyseal plate in this region exhibits slight elevation, and dysplasia beneath the medial trochlea reduces its volume, potentially explaining why a medial epiphyseal plate with a shorter anterior condyle has a smaller volume than a lateral plate with a shorter posterior condyle. Femoral torsion primarily arises from the distal femur.^5^ In patients with excessive DFT, abnormal epiphyseal plate development may lead to disproportionate growth between the posterior medial condyle and the posterior lateral condyle, and eventually increase the femoral torsion angle. Our study revealed significantly smaller medial epiphyseal plate volumes in patients with excessive DFT. Therefore, the anterior epiphyseal plate, which supports trochlear development, appears to play a substantial role in the remodelling process.

In early childhood, cartilage tissue accounts for most of the epiphysis and promotes the formation of distal femur morphology through endochondral osteogenesis and secondary ossification center growth. The stress on the cartilage and secondary ossification centre in the immature stage affects the development of bone and cartilage. We developed finite element models of the juvenile knee to analyze epiphyseal stress distribution, and demonstrated that altered patellofemoral alignment due to abnormal anatomical factors induced medial or lateral stress shifts in this region. Kura et al^35^ evaluated the bone transformation of tibial epiphysis by performing passive exercise training on mice, and found that exercise physiotherapy stimulates epiphyseal formation and restores bone integrity through osteoblast-mediated bone anabolic mechanism. This indicates that the epiphysis has a growth effect stimulated by cyclic mechanical stress. Our findings suggest that abnormal anatomical factors redirected stress anterolaterally toward the epiphysis, potentially stimulating growth at this region. In the rat animal model with femoral rotational osteotomy, stress stimulation in the anterolateral epiphyseal region compensated for bone loss resulting from reduced activity due to pain, whereas the anteromedial region exhibited significant bone loss from insufficient mechanical loading.

Patients with trochlear dysplasia exhibit developmental abnormalities in both the anterior and posterior condylar morphology. In our study, the posterolateral condyle constituted a low-stress region. As patellar dislocation and DFT shift stress anterolaterally, the posterolateral condyle remained a stress-deficient zone, potentially explaining its reduced size in trochlear dysplasia. Bone growth and ossification tend to occur at high-stress sites. This differential growth pattern of enhanced anterolateral development and restrained posterolateral growth likely contributes to distal femoral internal rotation and progressive torsion deformity during skeletal maturation. Therefore, the relationship between abnormal stress and torsion deformity does not demonstrate unilateral causation. Our findings indicate that these two pathological conditions exhibit mutual reinforcement.

In skeletally immature patients, surgical correction of recurrent patellar dislocation improves the epiphyseal morphology,^36^ and early surgical intervention enhances trochlear groove development.^37^ However, there are concerns regarding the use of derotational femoral osteotomy in skeletally immature populations,^38^ which limits opportunities for detailed longitudinal analysis. Rigorous longitudinal developmental follow-ups of conservatively treated patients are needed to elucidate the correlation between medial/lateral epiphyseal morphological changes and clinical outcomes. However, we believe that performing soft-tissue surgery alone to restore patellofemoral alignment and mechanical stress improves stress distribution in the bone-developing tissues and reverses the worsening of trochlear dysplasia.^39,40^ Given that trochlear dysplasia frequently coexists with multisegmental lower limb maldevelopment,^41^ full-length lower limb CT is routinely used to assess trochlear dysplasia. Building upon our CT-based epiphyseal plate mapping and 3D reconstruction image examination, we propose that epiphyseal plate imaging could serve as a routine supplemental examination for trochlear dysplasia. However, imaging-based anatomical studies have inherent limitations. Static representations cannot fully capture spatiotemporal functional dynamics, as these provide only structural foundations and spatial relationships without physiological validation. Future research should integrate anatomical and functional assessments, shifting the focus from observational descriptions to mechanistic investigations.

To investigate the relationship between femoral torsion and trochlear development, we induced patellar instability in the rat animal model by surgically increasing femoral torsion. Increased femoral torsion induced bone remodelling in the patellofemoral joint, increasing the patellar groove angle while reducing the patellar depth. These findings align with earlier observations in a rabbit model.^24^ Increased femoral torsion increases pressure on the lateral patellofemoral joint, which may induce abnormal morphological changes in the lateral femoral condyle and spur formation under excessive stress. Conversely, insufficient mechanical loading on the medial femoral condyle results in developmental impairment and progressive degeneration. Constructing the femoral torsion model outside the joint capsule reduced patellofemoral cartilage damage, more effectively revealing how stress alterations from bone deformities influence patellofemoral development.

Localized assessments of the subchondral bone regions revealed minimal changes in the lateral trochlea, whereas the medial trochlea showed pronounced bone loss. Further examination of the bone morphology adjacent to the distal femoral epiphyseal plate demonstrated substantial bone loss in the anterior medial epiphyseal region but minimal bone loss in the anterior lateral region. The subchondral bone morphology of the trochlea and epiphyseal region exhibited consistent patterns, indicating a potential relationship between trochlear structural changes and modifications in the femoral epiphyseal plate. Our model induced patellar instability through increased femoral torsion. The lateral trochlea maintained physiological pressure levels, supporting normal development, whereas the medial trochlea demonstrated bone loss resulting from inadequate stress stimulation. Osteoclast and osteoblast homeostasis becomes disrupted when mechanical stress exceeds or falls below cellular response thresholds, ultimately causing abnormal bone morphology.^42,43^ Further research is warranted to establish the mechanical stress threshold to sustain normal femoral condyle growth.

Endochondral ossification is a complex biological process that is essential for femoral development, and involves both cartilage growth and degradation alongside its gradual arthroplasty by bone tissue.^44^ Before femoral condylar bone tissue matures, the secondary ossification centre is surrounded by extensive cartilage that maintains continuity with the growth plate cartilage.^45^ Our findings indicate that cartilage primarily governed the morphological development of the femoral condyle and trochlea. However, the later emergence of secondary ossification centres, coupled with the advanced age of our animal model, precluded the examination of growth plate cartilage alterations. Future investigations should assess both growth plate cartilage and immature secondary ossification centers through integrated MRI and histological analysis.

This study has some limitations. First, although our power calculation confirmed that 98 knees was an adequate number for the correlation analysis, this single-centre retrospective study involved a small number of participants, potentially introducing selection bias during subject recruitment and data collection; multicentre studies with larger cohorts would strengthen these findings. Second, this exploratory study identified a correlation between the distal femoral epiphyseal plate and several bone deformities, however trochlear dysplasia typically involves multiple skeletal malformations. As the current findings cannot establish the primary factor associated with trochlear dysplasia, further aetiological investigation is warranted. While finite element models based on single individuals lack generalizability across populations, our individualized model integrated multiple pathological anatomical factors. These anatomical variations produced markedly distinct stress distribution patterns. Future research should develop cohort models encompassing diverse trochlear dysplasia types and varying degrees of rotational osteotomy.^46^ Third, in animal experiments, distance-based angle estimation during surgery is often imprecise. The analysis also lacked quantitative grouping of torsion angle variations. The design constraints of the implanted fixation devices prevented osteotomy in the femoral condylar region. Future studies should use larger animal models such as rabbits or pigs to more accurately replicate human skeletal development. Fourth, this study examined only the trabecular bone of the distal femoral epiphyseal plate, leaving the cartilage component unanalyzed. Subsequent research should integrate MRI with histological examinations to comprehensively assess the stratification and thickness of epiphyseal plate cartilage.

In conclusion, DFT alters stress distribution across the epiphysis and epiphyseal plate, which modifies the trabecular microarchitecture in both medial femoral condyles and anterior medial epiphyses, and results in different medial-to-lateral ratio of the distal femoral epiphyseal plate which indicates the severity of trochlear dysplasia, although genetic investigations are needed to establish its causality.

## Data Availability

The data that support the findings for this study are available to other researchers from the corresponding author upon reasonable request.
